# Conservation of proteins involved in oocyst wall formation in *Eimeria maxima*, *Eimeria tenella* and *Eimeria acervulina*

**DOI:** 10.1016/j.ijpara.2009.05.004

**Published:** 2009-08

**Authors:** Sabina I. Belli, David J.P. Ferguson, Marilyn Katrib, Iveta Slapetova, Kelly Mai, Jan Slapeta, Sarah A. Flowers, Kate B. Miska, Fiona M. Tomley, Martin W. Shirley, Michael G. Wallach, Nicholas C. Smith

**Affiliations:** a Institute for the Biotechnology of Infectious Diseases, University of Technology, Sydney, NSW 2007, Australia; b Nuffield Department of Pathology, University of Oxford, John Radcliffe Hospital, Oxford OX3 9DU, UK; c United States Department of Agriculture, Agricultural Research Service, Animal Parasitic Diseases Laboratory, Beltsville, MD 20705, USA; d Institute for Animal Health, Compton, Newbury, Berkshire RG20 7NN, UK

**Keywords:** *Eimeria*, Apicomplexa, Coccidiosis, Macrogametocyte, Oocyst wall, Immunolocalisation

## Abstract

Vaccination with proteins from gametocytes of *Eimeria maxima* protects chickens, via transfer of maternal antibodies, against infection with several species of *Eimeria*. Antibodies to *E. maxima* gametocyte proteins recognise proteins in the wall forming bodies of macrogametocytes and oocyst walls of *E. maxima*, *Eimeria tenella* and *Eimeria acervulina.* Homologous genes for two major gametocyte proteins – GAM56 and GAM82 – were found in *E. maxima, E. tenella* and *E. acervulina.* Alignment of the predicted protein sequences of these genes reveals that, as well as sharing regions of tyrosine richness, strong homology exists in their amino-terminal regions, where protective antibodies bind. This study confirms the conservation of the roles of GAM56 and GAM82 in oocyst wall formation and shows that antibodies to gametocyte antigens of *E. maxima* cross-react with homologous proteins in other species, helping to explain cross-species maternal immunity.

Maternal immunisation with proteins from the macrogametocytes of *Eimeria maxima* induces the production of antibodies that are transferred from hen to hatchling via the egg yolk and protect against infection (Wallach et al., 1995). Antibodies to these macrogametocyte proteins react with the wall forming bodies and the oocyst wall of *E. maxima* and have been used to chart the sequential formation of, first, the outer wall from wall forming bodies type 1 (WFB1) and, then, the inner wall from wall forming bodies type 2 (WFB2) (Ferguson et al., 2003).

Two gametocyte proteins from *E. maxima*, EmGAM56 and EmGAM82, have been studied in some detail. Both possess distinct regions with an unusually high prevalence of the amino acid tyrosine (Belli et al., 2002a,b, 2003a,b) and they each undergo processing into smaller peptides. These peptides are believed to be cross-linked via dityrosine bonding and, therefore, are thought to be crucial structural components of the mature oocyst wall (Belli et al., 2003a, 2006), helping to explain the remarkable resilience and resistance to chemical and environmental insults possessed by coccidian oocysts.

EmGAM56 and EmGAM82 are the major constituents of a commercially available subunit vaccine (CoxAbic^®^) against poultry coccidiosis, which has undergone extensive field testing worldwide (Wallach et al., 2008). Coccidiosis is a major veterinary problem in its own right but, perhaps more importantly, CoxAbic^®^ remains the first and only subunit vaccine against any protozoan disease to be commercialised and sold. One of the most intriguing features of the vaccine is its ability to cross-protect against every species of *Eimeria* tested to date (Wallach et al., 1995, 2008); this strikes a discordant note with what is known about the species-specific nature of immunity to coccidian and other parasites. We now provide three new lines of evidence to explain that cross-protection: (i) conservation of the function of GAM56 and GAM82 in oocyst wall formation across three species of *Eimeria* – *E. maxima*, *Eimeria tenella* and *Eimeria acervulina*; (ii) cross-reactivity of antibodies with the gametocyte proteins of these three species; and (iii) conservation of the genes for the gametocyte proteins across species. Additionally, we have constructed a preliminary epitope map of reactivity of protective antibodies with GAM56 and GAM82.

The development of sexual stages (micro- and macro-gametocytes) and formation of oocysts was observed within epithelial cells of the small intestine after infection with *E. maxima* (144 h p.i.) and *E. acervulina* (96 h p.i.) and of the caecum after infection with *E. tenella* (136 h p.i.). The gut samples were processed for immunofluorescence and immunoelectron microscopy and routine electron microscopy as described previously (Ferguson et al., 2003). Murine antibodies used in this study were those described previously: mouse anti-affinity purified gametocyte antigens (Ab2133, anti-EmAPGA; Wallach et al., 1990); mouse anti-recombinant *E. maxima* GAM56 (Ab2523, anti-rEmGAM56; Belli et al., 2003a); mouse anti-recombinant *E. maxima* GAM82 (Ab2733, anti-rEmGAM82; Belli et al., 2003b).

The development of macrogametocytes of *E. tenella* and *E. acervulina* was examined by electron microscopy and immunofluorescence, and compared to *E. maxima,* which has been described in detail previously (Ferguson et al., 2003). The resulting macrogamete in each case contains a centrally located nucleus with the cytoplasm containing numerous polysaccharide granules and lipid droplets with numerous peripherally located WFB1 and 2 and it was possible, for all species, to track the formation of the veil forming bodies (VFB) and WFB1 and 2. The VFB were identified by electron microscopy for both *E. tenella* and *E. acervulina* (not shown) and had the same distinctive appearance noted previously for *E. maxima* (Ferguson et al., 2003), being approximately 150–250 nm in diameter with electron lucent contents containing membrane-like fragments. Also, as described previously for *E. maxima* (Ferguson et al., 2003), the WFB1 were large membrane-bound vacuoles with electron dense contents that could be identified by both electron microscopy (not shown) and immunofluorescence (Fig. 1) in both *E. tenella* and *E. acervulina*. The WFB2, in both *E. tenella* and *E. acervulina,* were located within the rough endoplasmic reticulum, as has been observed in *E. maxima* (Ferguson et al., 2003), and consist of densely-packed spherical structures that have a honeycombed appearance (Fig. 1).

The WFBs of both *E. tenella* and *E. acervulina* were labelled with anti-EmAPGA (Fig. 1B and C) with a staining pattern similar to that observed in *E. maxima* (Fig. 1A). In immunostained sections of *E. tenella*, WFB2 also exhibited labelling with anti-rEmGAM56 (Fig. 1E) but were not labelled with anti-rEmGAM82 (not shown). In contrast, in *E. acervulina* the WFB2 labelled with both anti-rEmGAM56 (not shown) and anti-rEmGAM82 (Fig. 1F). For both *E. tenella* and *E. acervulina,* as for *E. maxima,* anti-rEmGAM56 and anti-rEmGAM82 did not stain the WFB1, which could be visualised by staining with Evans blue (Fig. 1D–F). All of these results were confirmed by immunogold electron microscopy (not shown) as described previously for *E. maxima* (Ferguson et al., 2003).

As described previously, the inner and outer layers of the oocyst wall in *E. maxima* stain with anti-EmAPGA (Fig. 1G) and a similar staining pattern was observed for oocysts of *E. tenella* (Fig. 1H) and *E. acervulina* (Fig. 1I). In *E. maxima,* both anti-rEmGAM56 (Fig. 1J) and anti-rEmGAM82 (not shown) stained the inner aspect of the inner layer but, in *E. tenella,* only anti-rEmGAM56 stained the inner aspect of the oocyst wall (Fig. 1K) and, in *E. acervulina,* the strongest staining was observed with anti-rEmGAM82 (Fig. 1L) with reduced staining with anti-rEmGAM56 (not shown). When examined by immunoelectron microscopy, sections stained with anti-EmAPGA showed numerous gold particles associated with the outer layer of the oocyst wall in both *E. tenella* and *E. acervulina* (not shown) as reported for *E. maxima* (Ferguson et al., 2003). In the case of *E. tenella* labelled with anti-rEmGAM56 and *E. acervulina* labelled with anti-rEmGAM82, the inner aspect of the inner layer and the ground substance between the plasmalemma of the cytoplasmic mass and the oocyst wall were labelled strongly (not shown), again as reported previously for *E. maxima* (Ferguson et al., 2003).

We next attempted to confirm cross-reactivity of specific antibodies against EmGAM56 and EmGAM82 with *E. tenella* and *E. acervulina.* Furthermore, we assessed whether similar processing of gametocyte proteins into smaller proteins takes place in *E. maxima, E. tenella* and *E. acervulina* as these three parasites transition from gametocyte to oocyst. In order to achieve these two aims, we collected samples of gametocytes, unsporulated oocysts and sporulated oocysts (Shirley, 1995; Wallach et al., 1989) and carried out SDS–PAGE and immunoblotting as described previously (Belli et al., 2002a).

In *E. maxima,* two predominant bands (∼58 kDa and ∼79 kDa) are recognised by anti-EmAPGA antibodies in gametocytes by Western blotting (Fig. 2), which correspond to the two major components of EmAPGA, EmGAM56 (migrating at ∼58 kDa; Belli et al., 2003a) and EmGAM82 (migrating at ∼79 kDa; Belli et al., 2003b). In *E. tenella*, anti-EmAPGA recognises a single polypeptide, presumed to be the homologue of EmGAM56 in gametocytes, albeit at a slightly smaller size, but does not recognise any polypeptides corresponding to EmGAM82. In *E. acervulina*, anti-EmAPGA recognises three predominant protein bands within a smear of protein that migrates >79 kDa (but note that elements of this smear can also be seen with the negative control sera). It is possible that the fastest migrating of these bands represents a protein homologue of EmGAM82. The antibodies do not recognise any proteins corresponding to the size of EmGAM56 in *E. acervulina*. In unsporulated and sporulated oocysts, anti-EmAPGA recognises a prominent 30 kDa protein band in *E. maxima* (Emwp30; Belli et al., 2003a), as well as a prominent ∼25 kDa band in *E. tenella*, which is possibly a homologue of Emwp30. Similarly, in *E. acervulina,* anti-EmAPGA shows weak reactivity with a ∼28 kDa protein band in unsporulated oocysts and a ∼20 kDa protein in sporulated oocysts. These results confirm and extend those described by Wallach et al. (1995) using chicken anti-EmAPGA antibodies, which also showed cross-reactivity of chicken antibodies to GAM56 and GAM82 across different *Eimeria* species and across gametocytes, sporulated and unsporulated oocysts.

A prominent band migrating at ∼58 kDa is recognised by anti-rEmGAM56 antibodies in *E. maxima* gametocytes corresponding to EmGAM56 (Belli et al., 2003a). This protein is also the predominant band recognised in *E. tenella*, again, migrating faster on the gels. A faint band is also detected at ∼50 kDa in gametocytes of *E. acervulina* and this could correspond to the EmGAM56 homologue. Again, as seen with anti-EmAPGA, a 30 kDa wall protein is recognised by anti-rEmGAM56 antibodies in unsporulated and sporulated oocysts of *E. maxima* and at 25 kDa in *E. tenella* oocysts. In *E. acervulina*, anti-rEmGAM56 antibodies recognise a ∼28 kDa protein band in unsporulated oocysts and a ∼20 kDa protein in sporulated oocysts, as described earlier for anti-EmAPGA. In *E. maxima*, weak reactivity of anti-rEmGAM56 was observed with a band migrating at ∼12 kDa in sporulated oocysts and was also detected with the same antibodies in *E. acervulina* sporulated oocysts. Whether these proteins represent the ∼14 kDa proteins described previously (Belli et al., 2003a; Eschenbacher et al., 1996) is yet to be determined. A prominent band migrating at ∼79 kDa, corresponding to EmGAM82, is recognised by anti-rEmGAM82 antibodies in *E. maxima* gametocytes (Belli et al., 2003b). The homologue is not detected in *E. tenella* and while very weak bands are observed in *E. acervulina* gametocytes, their identity is unclear.

A prominent band is recognised by anti-rEmGAM82 in *E. maxima* unsporulated and sporulated oocysts, migrating at ∼25 kDa protein. Once again, no cross-reactivity was observed with the homologue in *E. tenella*, while anti-rEmGAM82 recognised a ∼28 kDa protein band in unsporulated oocysts and a ∼20 kDa protein in sporulated oocysts of *E. acervulina*, as described earlier for anti-rEmGAM56 and anti-EmAPGA.

In summary, the overall staining patterns for anti-EmAPGA and anti-rEmGAM56 look similar for *E. maxima* and *E. tenella* but anti-rEmGAM82 recognition is more divergent for these two parasites and cross-reactivity is much less clear for gametocytes of *E. acervulina,* although there is relatively strong cross-reactivity in the oocyst stages of this parasite.

We next set out to discover whether homologous genes to *emgam56* and *emgam82* exist in *E. tenella* and *E. acervulina.* The gametocyte antigen homologues from *E. tenella* were retrieved by performing an omniBLAST search of finished and unfinished databases at the GeneDB *E. tenella* dedicated server (http://www.genedb.org/genedb/etenella/) using the following protein sequence queries: *emgam56* (**AAN05087**, Belli et al., 2002b) and *emgam82* (**AAO47083**, Belli et al., 2003b). Two homologous gene sequences, template 1 and template 2, encoding GAM56-like proteins (*etgam56 tmp 1* and *tmp2*) were identified within the *E. tenella* genome situated on contig EIMER_contig_00030093 (*E. tenella* contigs version 1-geneDB). The second *etgam56* sequence has also been reported by Krücken et al. (2007). However, in order to confirm that this unexpected finding is not simply a reflection of an incompletely annotated genome database, PCR was performed on *E. tenella* genomic DNA and cDNA isolated from merozoites (isolated from infected chicken caecae as described previously by Smith et al. (1994)), gametocytes, unsporulated and sporulated oocysts (as described by Shirley, 1995; Wallach et al., 1989). Thus, primers were designed to amplify the predicted coding regions from parasite cDNA. Total RNA was isolated from various *E. tenella* life cycle stages. PCR confirmed that the *etgam56* gene products were stage-specific and only amplified in gametocyte cDNA samples and genomic DNA samples (Fig. 3A). Products amplified from cDNA for *etgam56 tmp 1* and *2* were gel purified and sequenced (Australian Genome Research Facility, Queensland, Australia). Sequences were analysed using Lasergene^™^ v8.0 Sequence Analysis software (DNASTAR). Direct sequencing of these amplicons confirmed the presence of two unique *etgam56* genes that were identical to those identified in the genome database. In addition, a partial sequence of *E. acervulina* *gam56* (*eagam56*) was isolated from an *E. acervulina* cDNA library of merozoites purified 89 h p.i. (Miska et al., 2008) and an omniBLAST search of the *E. tenella* genome also revealed the presence of a predicted *E. tenella* *gam82* (*etgam82*) counterpart encoded on contig EIMER_contig_00031121 (*E. tenella* contigs version 1-geneDB). The retrieved protein sequences from blast searches were aligned using the ClustalW 1.83 program using BLOSUM (BLOcks of amino acid Substitution Matrix) (Chenna et al., 2003). The N-terminal signal peptide sequence was predicted using the SignalP 3.0 Server.

Both EtGAM56 TMP1 and TMP2 and EaGAM56 predicted proteins possess the general characteristics of EmGAM56 (Fig. 3B). It is evident that EtGAM56 TMP1 is most homologous to EmGAM56 (54% identity), followed by EaGAM56 (43%), which is missing the C-terminal portion of the protein (Fig. 3B). EtGAM56 TMP2 had the least homology to EmGAM56 (35%); it appears to be less homologous to its counterparts in several respects. First, the predicted N-terminal signal peptide comprises the first 22 amino acids in EtGAM56 TMP2 and 20 amino acids in the other homologues. Second, EtGAM56 TMP2 is significantly longer than its GAM56 counterparts and, third, it is significantly lower in tyrosine residues, with 20% less tyrosine than EmGAM56. The characteristic tyrosine-rich region of EtGAM56 TMP2, which divides the protein sequence into the N- and C-termini, is only 55 amino acids in length, compared with 74 amino acids and 77 amino acids of EmGAM56 and EtGAM56 TMP1, respectively. It is also evident that the N-terminal sequences have much greater sequence identity than the highly variable C-termini of the GAM56 proteins (Fig. 3B).

The EtGAM82 protein sequence is 42% identical to the EmGAM82 homologue (Fig. 3C). Although it appears that EtGAM82 is missing a part of the N-terminal sequence, it is clear that the predicted protein is highly conserved in its N-terminus. As seen with GAM56, the C-terminus of the protein is more divergent with less than 35% identity between the two species. Most importantly, the two unique tyrosine-rich regions characteristic of EmGAM82 are preserved in *E. tenella* (Fig. 3C).

Finally, we used truncated recombinant proteins (see Fig. 4) of EmGAM56 and EmGAM82 to construct a preliminary epitope map of these proteins. The truncated recombinant versions of EmGAM56 and EmGAM82 were constructed in the bacterial expression vector, pTrcHisB (Invitrogen), as described previously (Belli et al., 2002b, 2003b). Expression of the recombinant proteins was carried out following protocols described in the Xpress^™^ System, Protein Expression, pTrcHis manual (Invitrogen), as detailed previously (Belli et al., 2004) and the proteins purified from the supernatant under native conditions by nickel-nitrilotriacetic acid chromatography, as described in the protocols outlined in the QIA*expressionist* handbook for high-level expression and purification of 6xHis-tagged proteins (QIAGEN), as detailed previously (Belli et al., 2004).

The truncated recombinant *E. maxima* GAM56 proteins, rEmGAM56.469-840, rEmGAM56.172-840 and rEmGAM56.841-1052 were all expressed from pTrcHisB and were able to be purified by nickel-nitrilotriacetic acid chromatography to single protein species of appropriate masses, (21 kDa, 31.5 kDa and 13 kDa, respectively), confirmed by reactivity with anti-Penta•His antibodies (data not shown). The truncated N-terminal region, rEmGAM56.172-488, consistently failed to express. The purified recombinant proteins, together with rEmGAM56.172-1137 (described previously by Belli et al. (2004)) and EmAPGA, were used to coat ELISA plates (Costar) and then incubated with anti-APGA or normal chicken sera. Neither rEmGAM56.469-840 nor rEmGAM56.841-1052 reacted with anti-APGA whereas rEmGAM56.172-840 reacted at least as strongly as rEmGAM56.172-1137 with anti-EmAPGA (Fig. 4A). Thus, by a process of elimination, it can be concluded that immunodominant epitope(s) of EmGAM56 are located in the conserved amino-terminus of the protein.

The truncated recombinant *E. maxima* GAM82 proteins could not be expressed and purified as single protein species because each of these recombinant proteins are spontaneously degraded to a range of smaller products in situ (data not shown) making them unsuitable for use in ELISAs. Therefore, Western blotting was used to assess reactivity of the recombinant truncated proteins derived from EmGAM82 with anti-EmAPGA and normal chicken sera. The positive control, rEmGAM82.168-1887 (described previously by Belli et al. (2003b)), was recognised by the chicken anti-APGA serum as seen by the band at ∼80 kDa (Fig. 4B and C, lane 3). Numerous bands were apparent after probing with the negative control serum, the most intense being at ∼35 kDa, although another very faint band was observed at around 60 kDa (Fig. 4B and C). These bands were observed in all lanes suggesting non-specific binding. The chicken anti-EmAPGA serum did not recognise any other bands in the vector alone control (Fig. 4B and C lane 2). The serum did, however, react with a 65 kDa band representing rEmGAM82.168-1620 (Fig. 4B, lane 4). rEmGAM82.168-1169 was also recognised by the chicken anti-EmAPGA serum, with a clear band seen at 50 kDa (Fig. 4B, lane 5). rEmGAM82.168-824 was recognised by anti-EmAPGA, with a band at ∼37 kDa (Fig. 4C, lane 4) but, unfortunately, definitive identification of this truncated recombinant protein was hindered by a non-specific band at a similar molecular weight, recognised by normal chicken serum (Fig. 4C, lanes 6–8). Thus, as for EmGAM56, it can be concluded that immunodominant epitope(s) of EmGAM82 are located in the conserved amino-terminus of the protein.

It is clear from immunofluorescence and immunoelectron microscopy that the morphologies of macrogametes and oocysts, and the morphological transition from gamete to oocyst, are conserved across the species of *Eimeria* examined (*E. maxima, E. tenella* and *E. acervulina*) confirming and extending previous findings with *E. maxima* alone (Ferguson et al., 2003). Furthermore, homologues of *E. maxima* macrogamete proteins, EmGAM56 and EmGAM82, appear to be present in *E. tenella* and *E. acervulina*. Thus, antibodies raised against the *E. maxima* proteins cross-react with the WFBs within macrogametocytes of all three species, as shown by immunofluorescence and immunoelectron microscopy, and react in Western blots with proteins of various sizes in gametocyte and oocyst preparations from *E. maxima, E. tenella* and *E. acervulina*. Genes encoding homologous proteins were also identified in all three species (although a *gam82* gene has not yet been found in *E. acervulina*).

GAM56 and GAM82 homologues share much in common across the three species of *Eimeria* examined in this study. First, the relatively large proteins found in macrogametocytes of all three species are apparently processed into smaller proteins and incorporated into the inner wall of the oocyst. Second, all proteins share regions of richness of the amino acid, tyrosine, which is a key component of many diverse biological structures (Belli et al., 2006) including the oocyst wall of *E. maxima* (Belli et al., 2003a, 2006). Third, the highest region of homology between proteins from the three species is in the region at the amino terminus of the protein (i.e., prior to the tyrosine-rich region) and it is within this region that immunodominant epitopes of both EmGAM56 and EmGAM82 appear to localise. Taken together, these findings indicate a likely conservation of protein function at both the chemical level, where processing of precursor proteins into smaller peptides that form dityrosine crosslinks results in the production of the oocyst wall matrix, and at the morphological level, where a clear, ordered sequence of changes in structure and location of WFBs can be mapped (using the antibodies to the WFB proteins) as the macrogamete turns into an oocyst. Moreover, the mapping of the conserved epitopes for GAM56 and GAM82 to the pre-tyrosine amino terminus of the two proteins may provide a clue as to how antibodies generated in response to maternal immunisation with purified gametocyte antigens interfere with oocyst development; this finding may indicate that antibodies “protect” GAM56 and GAM82 from degradation to the smaller tyrosine-rich proteins that are incorporated into the oocyst wall (by contrast, if the antibodies had reacted with the tyrosine-rich regions, it would be argued that the effects of immunisation are felt further downstream and that protective antibodies interfere with the cross-linking of the smaller tyrosine-rich proteins).

Although the shared characteristics of the gametocyte proteins of *E. maxima, E. tenella* and *E. acervulina* are considerable, there are differences apparent as well. Most notably, the sizes of the protein bands recognised in the different species are variable, although this is perhaps not too surprising given observations of diverse sizes of proteins incorporated into the oocyst wall after processing of EmGAM56 and EmGAM82 (Belli et al., 2003a). However, whilst EmGAM56 and EmGAM56 oocyst wall derivatives are recognised by both anti-EmAPGA and anti-rEmGAM56 antibodies in *E. maxima and* *E. tenella* gametocytes and oocysts, EmGAM82 and EmGAM82 oocyst wall derivatives are not recognised by anti-EmAPGA or anti-rEmGAM82 antibodies in *E. tenella* gametocytes, even though a homologous gene has been found in *E. tenella*. This may indicate that the epitope(s) recognised in EmGAM82 are not identical to those in the *E. tenella* protein, an argument that is given further strength by the relatively weak immunofluorescence reactivity of anti-EmGAM82 antibodies to *E. tenella* gametocytes and oocysts. While the degree of cross-reactivity of these antibodies in *E. acervulina* gametocytes is similarly not clear, they do cross-react with oocyst wall proteins that migrate at similar molecular weights to the GAM56 and GAM82 wall derivatives. These findings might suggest that, while the oocyst wall precursor proteins are conserved to some degree in the gametocytes, once processed during wall formation, the oocyst wall proteins adopt a structural epitope in the wall that is more easily recognised.

In summary, we have demonstrated that homologues of the *E. maxima* macrogamete proteins, EmGAM56 and EmGAM82, exist in *E. tenella* and *E. acervulina* and share regions of great richness of tyrosine. Furthermore, these precursor proteins appear to be similarly processed into smaller peptides in all three species and are incorporated into the inner oocyst wall. The oocyst wall plays a vital role in protecting the parasite while in the external environment and requires very distinct mechanical and chemical characteristics. It is possible that these requirements constrain the molecular variability, resulting in a basic similarity in molecular structure. This could explain the cross-reactivity between species and why a vaccine based on purified gametocyte antigens of one species may be able to provide cross-species protection. Thus, the cross-reactivity of anti-APGA antibodies and the conservation of genes, especially the high homology that exists in the region of the genes that codes for the dominant epitope(s) of the proteins, helps to explain the cross-protection that results from the maternal transfer of antibodies after vaccination of hens with purified gametocyte antigens. This provides an immunological basis of action for this mode of vaccination.

## Figures and Tables

**Fig. 1:**
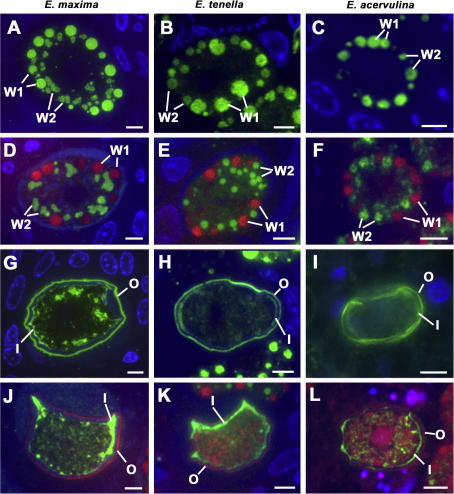
Details of mature macrogametes (A–F) or developing oocysts (G–L) immuno-stained with anti-EmAPGA (antibody to *Eimeria maxima* Affinity Purified Gametocyte Antigens) (A–C, G–I), visualised with FITC (green) and counter-stained with DAPI (blue) or anti-rEmGAM56 (antibody to recombinant *E. maxima* 56 kDa gametocyte protein) (D, E, J, K) or anti-rEmGAM82 (antibody to recombinant *E. maxima* 82 kDa gametocyte protein) (F, L) visualised with FITC and counter-stained with DAPI (blue) and Evans blue (red). The first column (A, D, G, J) shows examples of *E. maxima*, the second column (B, E, H, K) is *Eimeria tenella* and the third column (C, F, I, L) is *Eimeria acervulina.* Bars represent 5 μm. The anti-EmAPGA labels the WFB1 (Wall Forming Body Type 1) (W1) and WFB2 (W2) in all three species (A–C) while anti-rEmGAM56 labels the WFB2 (W2) but not the WFB1 (W1) in *E. maxima* (D) and *E. tenella* (E) and anti-rEmGAM82 shows a similar staining pattern in *E. acervulina* (F). In the oocysts, anti-EmAPGA labels the outer layer (O) and the inner layer (I) of the oocyst wall in all three species (G–I). In contrast, anti-rEmGAM56 in *E. maxima* (J) and *E. tenella* (K) and anti-rEmGAM82 in *E. acervulina* (L) stain the inner layer (I) but not the outer layer of the oocyst wall (© David J.P. Ferhuson).

**Fig. 2:**
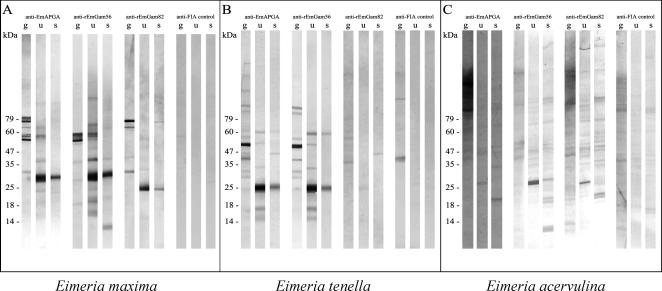
Cross-reactivity of antibodies to *Eimeria maxima* gametocyte antigens with *E.maxima* (A) *Eimeria tenella* (B) and *Eimeria acervulina* (C)*.* Gametocyte (g), unsporulated oocysts (u) and sporulated oocysts (s) were separated by SDS–PAGE, transferred to Immobilon-P membrane and probed with antisera to *E. maxima* gametocyte proteins (anti-EmAPGA [antibody to *Eimeria maxima* Affinity Purified Gametocyte Antigens] anti-rEmGAM56 [antibody to recombinant *E. maxima* 56 kDa gametocyte protein], anti-rEmGAM82 [antibody to recombinant *E. maxima* 82 kDa gametocyte protein]) or antisera from animals immunised with Freund’s Incomplete Adjuvant (FIA) plus saline. Bands were visualised with SIGMA^FAST™^ BCIP/NBT after incubation with secondary antibody. Parasite loadings per well were as follows: *E. maxima –* 2 × 10^3^ gametocytes, 3 × 10^3^ unsporulated oocysts, 2.5 × 10^4^ sporulated oocysts; *E. tenella* – 2 × 10^3^ gametocytes, 2 × 10^3^ unsporulated oocysts, 2 × 10^4^ sporulated oocysts; *E. acervulina* – 7.5 × 10^3^ of gametocytes, 6 × 10^4^ unsporulated oocysts, 2.4 × 10^5^ sporulated oocysts.

**Fig. 3:**
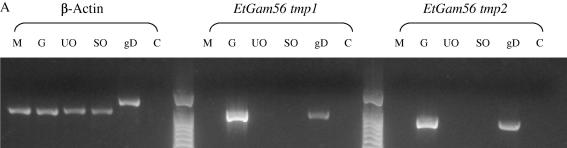

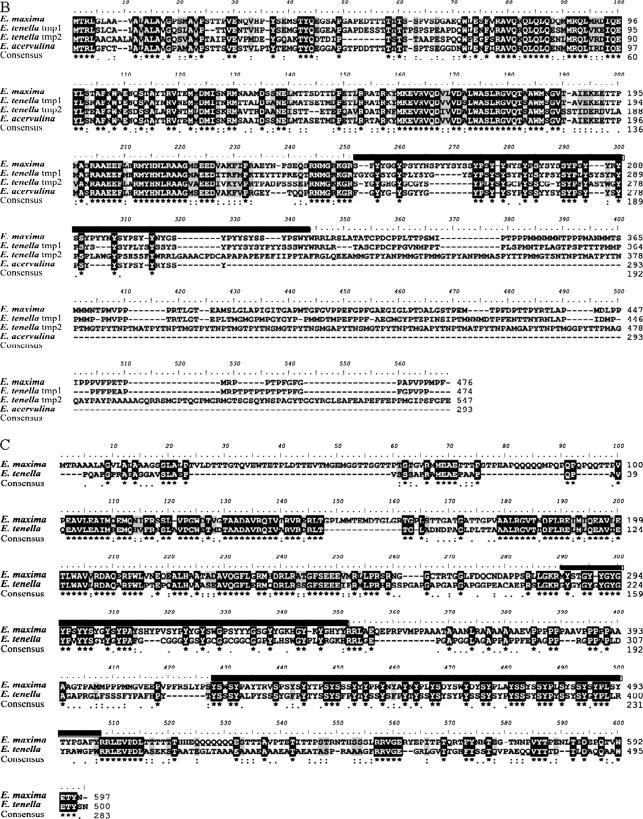
Stage-specific gene expression of the *gam56* gene in *Eimeria tenella* (A) and multiple protein sequence alignment of GAM56 (56 kDa gametocyte protein) (B) and GAM82 (C) from *Eimeria maxima, E. tenella* and *Eimeria acervulina*. Total RNA was extracted from *E. tenella* life cycle stages, cDNA synthesised and parasite genes amplified by PCR (A). Purified merozoites (1 × 10^7^) and gametocytes (1 × 10^6^) were resuspended in 1 ml TRIzol^®^ Reagent and homogenised by pipetting. Unsporulated oocysts (2 × 10^5^) and sporulated oocysts (5 × 10^5^) were resuspended in 1 ml TRIzol^®^ Reagent and 1 vol. of glass beads was added and the sample was vortexed for 1 min intervals until disruption of oocysts was confirmed by bright field microscopy. All TRIzol^®^ treated samples were left at room temperature for 10 min and total RNA isolated by chloroform extraction and isopropanol precipitation. RNA was quantified using a NanoDrop^™^ ND-1000 Spectrophotometer and cDNA was synthesised using SuperScript^™^ III Reverse Transcriptase (Invitrogen) according to the manufacturer’s instructions. Parasite cDNA samples were standardised by quantification of *E. tenella* β-actin PCR product. Forward primer E0043 (5′ ggaattcgttggccgcccaagaatcc 3′) and reverse primer E0044 (5′ gctctagattagctcggcccagactcatc 3′) were used to generate a 1020 bp β-actin cDNA PCR product. Forward primer E0030 (5′ catatggtggagaacacggtgcac 3′) and reverse primer E0031 (5′ ctcgagttagtaccagctggaggagta 3′) were designed to amplify a 906 bp cDNA product of *etgam56 tmp 1*. Forward primer E0035 (5′ catatggtagaagtgccaatggac 3′) and reverse primer E0036 (5′ cacgtgttagtagaagctggagtggct 3′) were designed to amplify an 804 bp cDNA product of *etgam56 tmp 2*. Each PCR reaction contained 50 ng of parasite stage-specific cDNA, 0.2 μM forward primer, 0.2 μM reverse primer, 1 × AccuPrime™ reaction mix, and AccuPrime^™^*Pfx* DNA polymerase (Invitrogen). The PCR reaction was carried out as follows: initial denaturation 95 °C for 3 min; 95 °C for 30 s; 61 °C for 1 min; 68 °C for 1.5 min, for 25 cycles with a final extension at 68 °C for 10 min. Merozoite (M), gametocyte (G), unsporulated oocyst (UO), sporulated oocyst (SO), genomic DNA control (gD) and a negative control sample with no template (C) were electrophoresed on a 1% agarose gel alongside a 100 bp ladder (Invitrogen) and visualised using Gel Red™ (Biotium). The β-actin gene product (∼1000 bp) was amplified from cDNA from all *E. tenella* life cycle stages. The *etgam56 tmp 1* gene product (∼900 bp) and *etgam56 tmp 2* gene product (∼800 bp) were amplified from cDNA of *E. tenella* gametocytes and the genomic DNA control only. The amino acid sequences of GAM56 and GAM82 were aligned using the program Clustal X and BLOSUM (BLOcks of amino acid Substitution Matrix) (Chenna et al., 2003) (B and C). Tyrosine rich regions (highlighted with a black line above the alignment) split the protein sequences into the N- and C-terminal ends. Symbols on the lowermost Clustal consensus lines represent amino acid positions: ‘∗’ fully conserved, ‘:’ one of the strong groups of amino acids is conserved, and ‘.’ one of the weak amino acid groups is conserved.

**Fig. 4:**
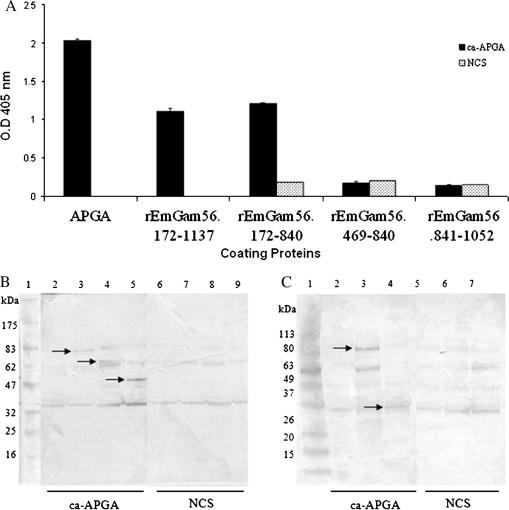
Analyses of the antigenicity of truncated recombinant proteins of *Eimeria maxima* GAM56 (EmGAM56; *E. maxima* 56 kDa gametocyte protein) (A) and EmGAM82 (*E. maxima* 82 kDa gametocyte protein) (B, C). The recombinant versions of *emgam56 (*designated *remgam56.172-1137* here) and *emgam82* (designated *remgam82.168-1887* here) described by Belli et al. (2002b, 2003b, 2004) served as parent constructs for DNA amplification of various regions by PCR. DNA fragments were amplified from the parent construct using specific primer sets and designated according to the first and last base pairs included in their sequences: *remgam56.172-840* (forward primer = 5′ cgcggatccgaccactcctgtggagaatcaggt 3′, reverse primer = 5′ cgcgaattcgatcatgtccatcatctcggtaac 3′); *remgam56.469-840* (forward primer = 5′ cgcggatccgtccaacagaatgaatgcagcaat 3′, reverse primer = 5′ ggcgaattcgctcctgccctttctgcccatatt 3′); *remgam56.172-840* (forward primer = 5′ cgcggatccgaccactcctgtggagaatcaggt 3′, reverse primer = 5′ ggcgaattcgctcctgccctttctgcccatatt 3′); *remgam56.841-1052* (forward primer = 5′ cgcggatccgttctactcctgcggctatcccag 3′, reverse primer = 5′ cgcgaattcgctggggtagctgctataactgta 3′); *remgam82.168-1620* (forward primer = 5′ cgcggatcctactgtattggacacaacgactggc 3′, reverse primer = 5′ cgcgaattcatcagggacctctagtctttctataaaagg 3′); *remgam82.168-1169* (forward primer = 5′ cgcggatcctactgtattggacacaacgactggc 3′, reverse primer = 5′ cgcgaattcgcataacaggtcttggttcctgctc 3′); and *remgam82.168-824 1169* (forward primer = 5′ cgcggatcctactgtattggacacaacgactggc 3′, reverse primer = 5′ ggcgaattcgcacgaagacgatcatgcatgcga 3′). Purified recombinant proteins of EmGAM56 were coated onto ELISA plates (100 ng/well) and exposed to 100 μl of chicken anti-EmAPGA (*E. maxima* Affinity Purified Gametocyte Antigens) serum (ca-APGA; 1:100 dilution) or negative control chicken serum (NCS) and assayed using methods described previously (Belli et al., 2004) (B). Results are the means of duplicate assays. For EmGAM82, induced recombinant bacterial lysates were separated by SDS–PAGE and proteins transferred to polyvinylidene fluoride membrane and probed with chicken anti-EmAPGA serum (ca-APGA), and negative control chicken serum (NCS) using protocols described previously (Belli et al., 2004) (B and C). Lanes for (B) are: (1) protein molecular weight markers; (2 and 6) bacterial lysate of induced pTrcHisB vector; (3 and 7) bacterial lysate of induced *remgam82.168-1887* in pTrcHisB; (4 and 8) bacterial lysate of induced *remgam82.168-1620* in pTrcHisB; (5 and 9) bacterial lysate of induced *remgam82.168-1169* in pTrcHisB. Lanes for (C) are: (1) benchmark prestained protein molecular weight markers; (2 and 5) bacterial lysate of induced pTrcHisB vector; (3 and 6) bacterial lysate of induced *remgam82.168-1887* in pTrcHisB; (4 and 7) bacterial lysate of induced *remgam82.168-824* in pTrcHisB.
